# Atypical Neurological Manifestation in Childhood Microscopic Polyangiitis: A Case Report and Review of Literature

**DOI:** 10.3389/fped.2022.855338

**Published:** 2022-03-11

**Authors:** Preawkalaya Suksai, Suphawe Wasuanankun, Vitit Lekhavat, Ornatcha Sirimongkolchaiyakul, Sirikarn Tangcheewinsirikul

**Affiliations:** ^1^Department of Pediatrics, Faculty of Medicine Vajira Hospital, Navamindradriraj University, Bangkok, Thailand; ^2^Department of Radiology, Faculty of Medicine Vajira Hospital, Navamindradriraj University, Bangkok, Thailand; ^3^Division of Nephrology, Department of Pediatrics, Faculty of Medicine Vajira Hospital, Navamindradriraj University, Bangkok, Thailand; ^4^Division of Rheumatology, Department of Pediatrics, Faculty of Medicine Vajira Hospital, Navamindradriraj University, Bangkok, Thailand

**Keywords:** CNS vasculitis, microscopic polyangiitis (MPA), children, myocarditis, rapidly progressive glomerulonephritis

## Abstract

Microscopic polyangiitis (MPA), a systemic necrotizing vasculitis of small vessels, is primarily associated with necrotizing and crescentic glomerulonephritis and pulmonary capillaritis. Neurological involvement, particularly of the central nervous system (CNS) is scarcely observed. The diversity of CNS symptoms could puzzle the diagnosis causing delays in treatment and potentially having a considerable effect on patient's quality of life or even death. The aim of this case report is to highlight the unusual manifestation of MPA in order to raise awareness of this orphaned disease among pediatricians or even pediatric rheumatologists and neurologists.

**Case Report:**

Herein we report the case of a 13-year-old Thai girl diagnosed with MPA presented with rapidly progressive glomerulonephritis (RPGN). Renal biopsy was performed demonstrated crescentic glomerulonephritis with negative immunofluorescence and positive titer of myeloperoxidase (MPO) antibody. Pulse methylprednisolone (MP) and cyclophosphamide (CYC) as well as plasmapheresis were initiated. Despite treatment with prednisolone (45 mg/day) and monthly CYC for two doses, she experienced a brief generalized tonic–clonic seizure during the follow-up period. The potential differential diagnosis of new-onset neurological manifestation contains infection owing to the immunocompromised status of the patient and CNS vasculitis as a result of the disease itself. Lumbar puncture was performed, and cerebrospinal fluid analysis demonstrated pleocytosis with negative infectious panel. Contrast magnetic resonance imaging studies of the brain showed multifocal patchy T2/FLAIR-hyperintense lesions in the cerebral as well as cerebellum regions, and irregular narrowing along the V4 segment of the right vertebral artery was demonstrated in magnetic resonance angiography. In the presence of CNS vasculitis, pulse MP and CYC were provided. The symptom of nervous system has progressively improved.

**Conclusion:**

In our case, MPA revealed RPGN with neurological manifestation. Despite the fact that it is scarcely reported, CNS vasculitis is one of the organ-threatening symptoms. To improve patient morbidity and mortality, multidisciplinary care teams with prompt diagnosis and treatment are highly recommended.

## Introduction

Microscopic polyangiitis (MPA) is one of the most common types of antineutrophil cytoplasmic antibody (ANCA) (1)–associated small-vessel vasculitis. Neurological involvement, particularly of the central nervous system (CNS), is infrequently seen but seem to have significant morbidity and mortality in cases of MPA (2), particularly in children. Herein we present the case of a Thai girl diagnosed with MPA and showing atypical neurological manifestation. However, the aim of this case report is to highlight the unusual symptom of MPA in order to raise awareness of this orphaned illness among pediatricians and care professionals.

## Methods

We searched the MEDLINE/Pubmed databases from the year of creation until January 2021. The multiple search Medical Subject Headings (MeSH) terms were systemically used alone or in combination: “Central Nervous System,” “Vasculitis,” “Microscopic Polyangiitis,” “Child” and their derivatives (the full search strategy can be found in Supplementary Material). We also searched the reference lists of all included publications for further potential studies. Six articles describing children diagnosed with MPA with CNS manifestation were found during the literature search.

## Case Description

A 13-year-old, previously healthy girl was transferred to Vajira Hospital with high-grade fever, edema, and progressive dyspnea for 3 weeks. Physical examination revealed hypertension, pale conjunctiva, puffy eyelids, and pitting edema in both legs. Cutaneous lesions or hemoptysis was not present. Neurological examination was unremarkable. Laboratory investigations revealed the following: 80 mg/dL blood urea nitrogen, 9.62 mg/dL serum creatinine, and 7.0 mL/min/1.73 m^2^ estimated glomerular filtration rate according to the Schwartz formula. Urine analysis showed microscopic hematuria with dysmorphic red blood cells and urine protein/creatinine ratio (UPCR) of 1.96. Complete blood count revealed anemia with hemoglobin levels of 4.7 g/dL; white blood cell and platelet counts appeared normal.

**Figure 1:**
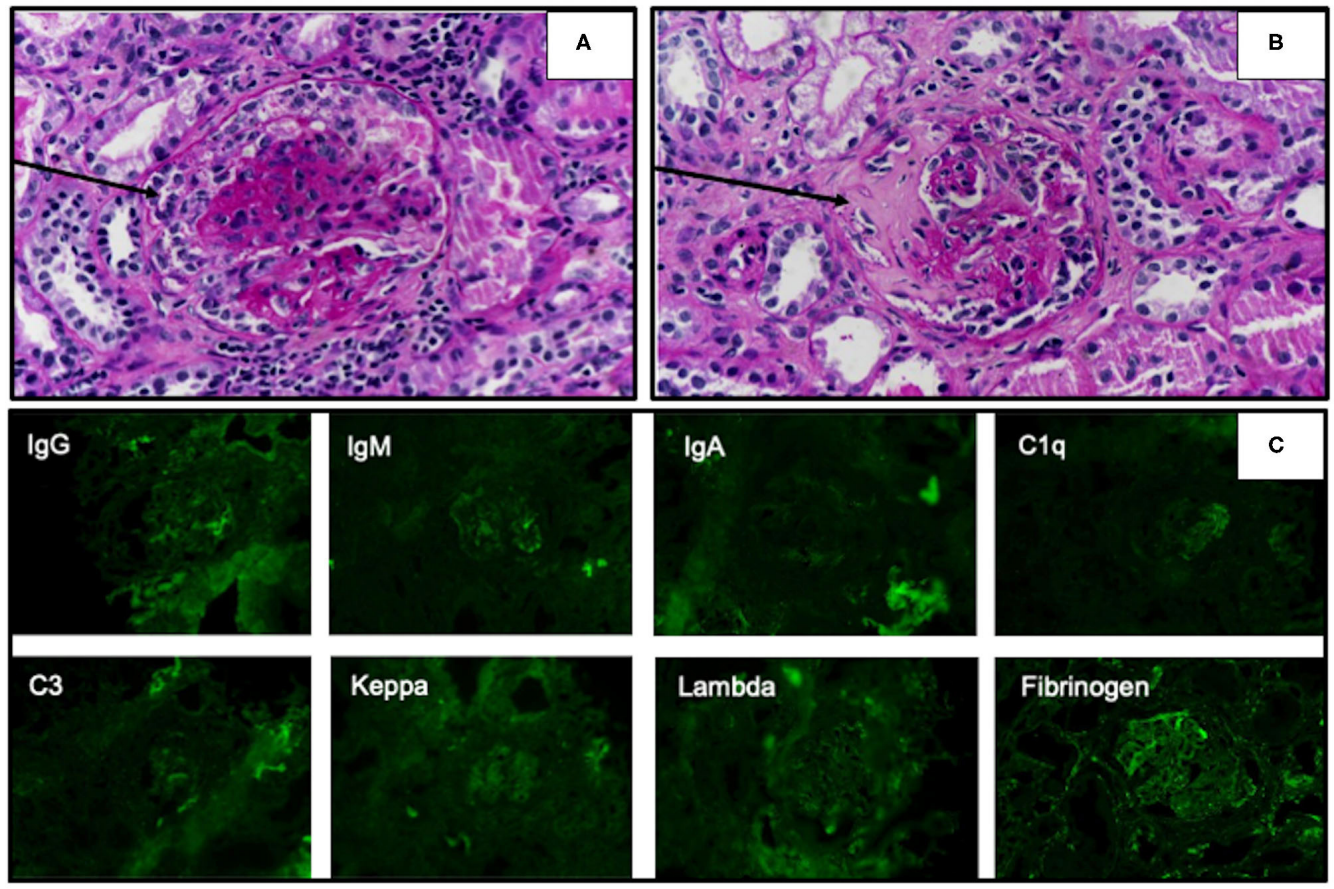
Light microscopy of renal biopsy specimens stained with the periodic acid–Schiff technique showed cellular crescentic formation (**A**, arrow) and fibrocellular crescent in Bowman's space (**B**, arrow). Immunofluorescence studies revealed negative staining of all complements and immunoglobulins **(C)**.

**Figure 2:**
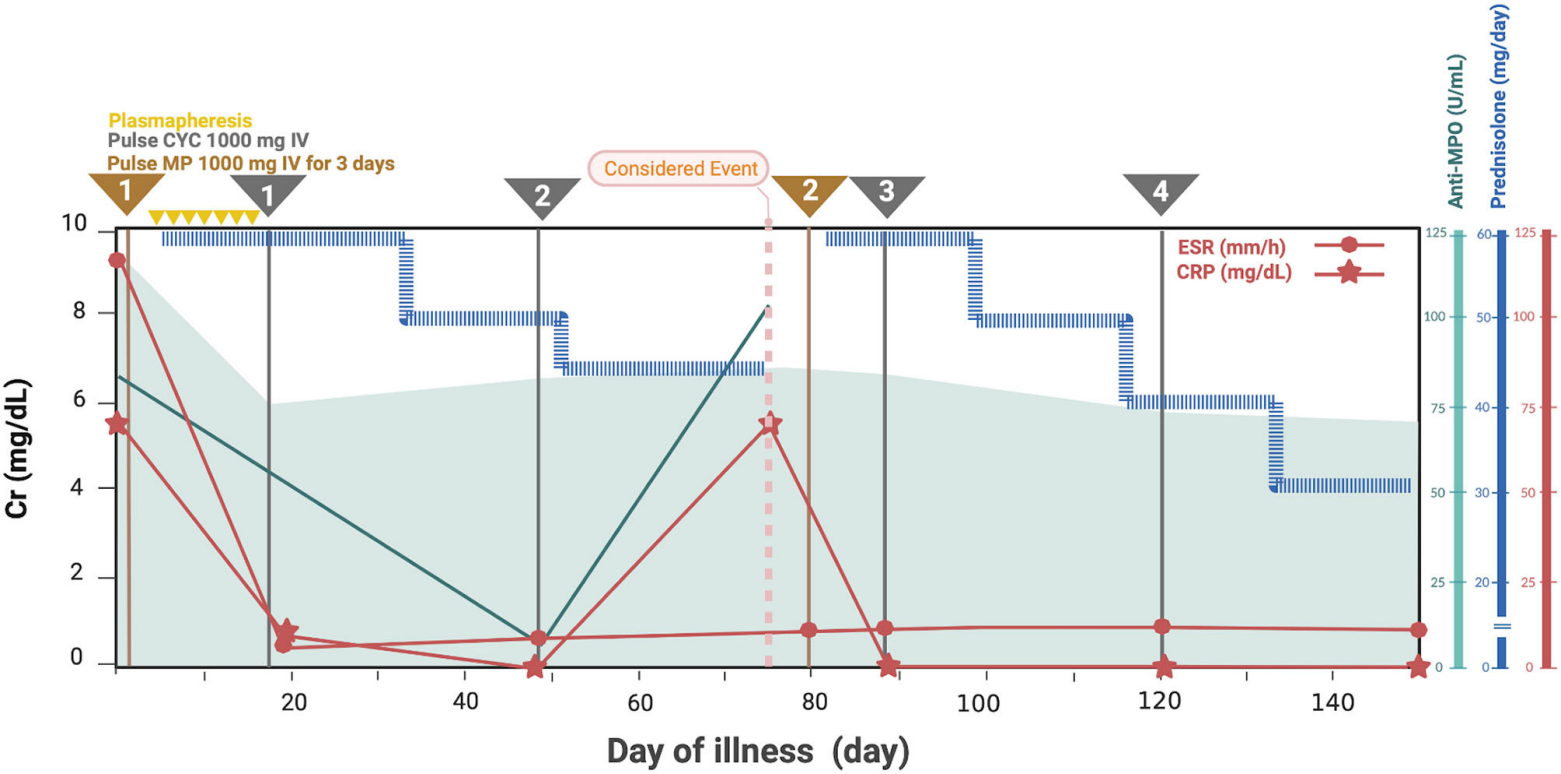
Clinical manifestation, essential laboratory findings, and management of the patient. Cr, creatinine; CRP, C-reactive protein; CYC, cyclophosphamide; ESR, erythrocyte sedimentation rate; MP, methylprednisolone; MPA, microscopic polyangiitis; MPO, myeloperoxidase.

Based on her symptoms, rapidly progressive glomerulonephritis was suspected. Ultrasonography of the kidney, ureter, and bladder system showed normal kidney size with diffuse bilateral renal echogenicity. On further examination, we found that erythrocyte sedimentation rate (119 mm/h) and C-reactive protein (66 mg/dL) levels were increased, and MPO antibody titer was also elevated at 76 U/mL (0–10 U/mL). Levels of complements and immunologic markers appeared normal: 1.13 g/L C3 (0.9–1.8 g/L), 28 mg/dL C4 (10–40 mg/dL), 6.15 IU/mL antidouble-stranded DNA antibody (0–15 IU/mL), and 0.8 IU/mL anti-Sm antibody (0–10 IU/mL). Renal biopsy demonstrated crescentic glomerulonephritis with negative immunofluorescence (Figure 1), which led to the diagnosis of MPA. Pulse methylprednisolone (MP) 1,000 mg for 3 consecutive days and cyclophosphamide (CYC) 1000 mg (775 mg/m^2^/dose) intravenous as well as plasmapheresis were initiated, along with emergency hemodialysis and antihypertensive drug administration. As a consequence, her fever, generalized edema, and dyspnea had subsided. Anti-MPO was returned normal at 7.1 U/mL (0-10 U/mL), however her renal function had not recovered as described in timeline diagram (Figure 2).

**Figure 3:**
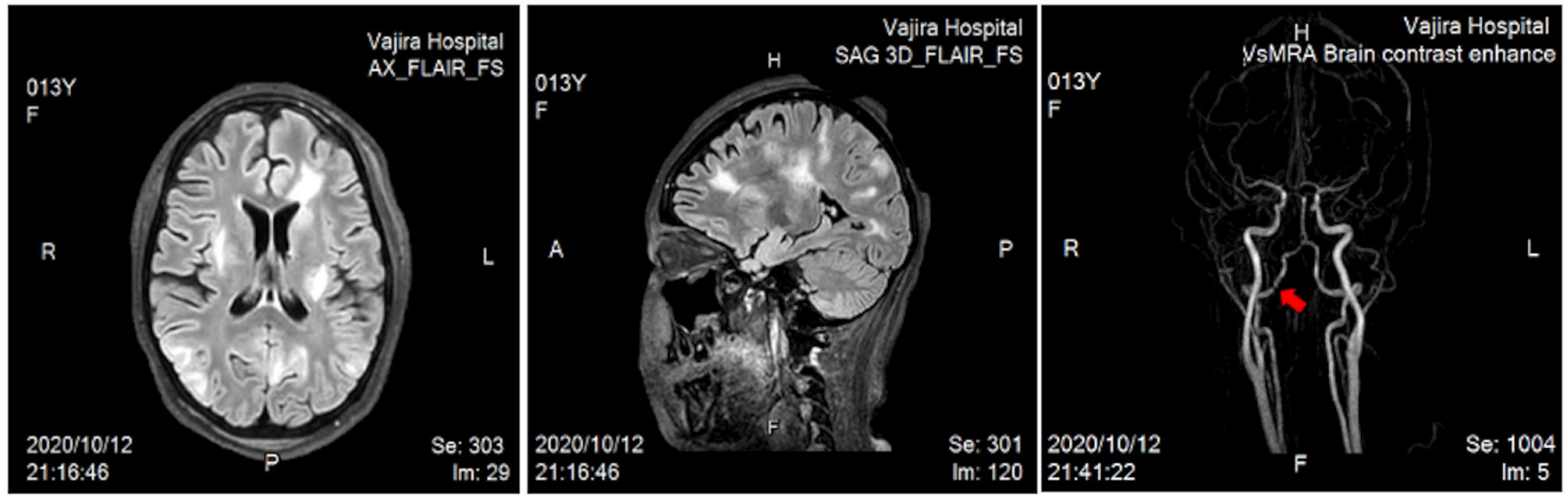
Magnetic resonance imaging of the brain revealed multifocal patchy T2/FLAIR-hyperintense lesions in the subcortical and deep white matter of the bilateral fronto–parieto–occipito–temporal lobes, posterior limb of the left internal capsule, and superior cerebellar vermis. Bilateral ACAs, MCAs, and PCAs were unremarkable, but magnetic resonance angiography showed irregular narrowing along the V4 segment of the right vertebral artery (arrow). ACAs, anterior cerebral arteries; MCAs, middle cerebral arteries; PCAs, posterior cerebral arteries.

Ten weeks after the onset, the patient consequently experienced a brief generalized tonic–clonic seizure with both eyes deviating to the right. Her blood pressure was 153/103 mmHg and she had a fever of 38°C for a few days despite being on prednisolone 45 mg/day (1.2 mg/kg/day), pulse CYC 1,000 mg monthly, and hemodialysis two times per week. During the time, she had minimal urine and her renal function revealed as following: 95 mg/dL blood urea nitrogen, 7.15 mg/dL serum creatinine, and 8 mL/min/1.73 m^2^ estimated glomerular filtration rate according to the Schwartz formula. Urine examination showed significant albuminuria and active sediments comprising red blood cells 50–100/hpf, white blood cells 2-3/hpf and frequent granular casts. UPCR was of 1.76. Differential diagnoses included posterior reversible encephalopathy syndrome, meningoencephalitis, and MPA with CNS involvement. Lumbar puncture was performed, and magnetic resonance imaging (MRI) and magnetic resonance angiography (MRA) of the brain were conducted. Cerebrospinal fluid analysis revealed pleocytosis with negative infectious panel. Her blood glucose and serum electrolytes were normal. Further, contrast MRI studies of the brain showed multifocal patchy T2/FLAIR-hyperintense lesions in the cerebral as well as cerebellum regions, and MRA revealed irregular narrowing along the V4 segment of the right vertebral artery (Figure 3). At the time of admission to the pediatric intensive care unit, her resting heart rate increased from 60–70 to 130–140 beats/min, and clinical dyspnea and edema were observed. Besides, the levels of following cardiac biomarkers were elevated: 40 U/L myocardial band of creatine kinase, >35,000 pg/mL probrain natriuretic peptide, and 577 ng/L troponin T. To investigate acute myocarditis, an experienced pediatric cardiologist performed echocardiography, which indicated a moderate degree of mitral regurgitation with minimal pericardial effusion and no coronary abnormalities (left ventricular ejection fraction, 30% and cardiac index, 2.5 L/min/m^2^). The possible infectious causes of myocarditis were negative, which led to the diagnosis of acute myocarditis in MPA.

Pulse MP and CYC were administered considering the presence of CNS vasculitis and acute myocarditis. For cardiovascular support, inotropic drugs and oxygen therapy were initiated. Outpatient follow-up, her resting heart rate returned to normal; further, echocardiograph showed 56% left ventricular ejection fraction, trivial mitral regurgitation, and mild aortic regurgitation. The renal function was stable at end-stage renal disease and the modality of replacement was shifted to peritoneal dialysis due to the patient's and family's preference. Neurological symptoms in terms of consciousness improved gradually, and there were no seizures while on stable antiepileptic drug; moreover, electroencephalograms taken 2 weeks and 2 months following treatment revealed an improvement in encephalopathy, with no new symptoms and/or signs after 2 months.

## Discussion

ANCA-associated systemic vasculitis (AASV) targets medium and particularly small vessels, leading to end-organ inflammation. According to the classification by the Chapel Hill Consensus 2012 for systemic vasculitis, AASV can be classified into granulomatosis with polyangiitis (GPA), eosinophilic granulomatosis with polyangiitis (EGPA), and MPA (1). GPA is generally characterized by the presence of antibodies to proteinase 3, while the other two types mainly show the presence of antibodies to MPO. Related to the adult-onset of AASV, childhood-onset GPA is the most prevalent type, followed by MPA and EGPA. Epidemiological documents of AASV are inadequately characterized, with estimated prevalence of 3.41–4.28 per million children (9), due to the limited classification criteria. The overall incidence of AASV is between 13 and 20 per million population (10). Owing to the limited classification criteria for MPA and its rarity, the global incidence of childhood MPA is underreported. Less is known about the incidence of MPA in pediatric population, in the Swedish study have suggested that the rate of incidence of MPA in children is 1.4 per million per year (11). Common findings among children with AASV include fever and upper airway involvement, particularly nasal cartilage damage. Furthermore, recurrent rate and damage accrual are reported to be more severe in children with AASV (12). Disease onset is particularly seen in the second decade of life, and disease prevalence is more predominant in females (13).

The etiology and pathogenicity of AASV remain unclear. Several factors evidently contribute to disease precipitation, including genetic susceptibility, epigenetic variables, and environmental factors, such as exposure to antithyroid medications as well as silica dust; in addition, impairment of neutrophil extracellular traps is reportedly associated with AASV (14).

In >70% patients with MPA, the kidney is the most commonly affected organ, with one-third patients exhibiting rapidly progressive glomerulonephritis at the time of diagnosis (3), which was also observed in our case. Constitutional symptoms, fever, and fatigue are also seen. Pulmonary symptoms, such as hemoptysis, cough, pulmonary vasculitis, and pulmonary hemorrhage, have also been reported in cases of childhood MPA. In comparison to renal and pulmonary symptoms, neurological symptoms such as seizures are observed in only 5% patients (13).

A wide range of neurological manifestations can occur, including peripheral nervous system involvement with, for example, mononeuritis multiplex and polyneuropathy, as reported in two patients by Iudici et al. (15) and also CNS involvement (see Table 1). Cognitive decline, headache, seizure, visual disturbance, cerebrovascular disease, and spinal cord complaints are all manifestations of CNS involvement in AASV (16). In a Japanese and Chinese retrospective cohort study, three children with MPA were reported to experience seizures and had an unfavorable outcome (3, 6). Although difficult to evaluate, especially in children, cognitive deterioration was observed in around one-third of patients with AASV (17), impacting abstract thinking, attention, and nonverbal memory. The authors also suggest recognizing a decline in academic performance in addition to comprehensive perform neurological examination. On neuroimaging of AASV patient, multiple white matter lesions along the periventricular or juxtacortical regions are frequently associated with cognitive impairments (17). Bhadu et al. (8) reported the case of an Indian patient with childhood MPA who presented with seizure and her MRI of the brain revealed diffuse brain parenchymal involvement together with multistage of microhemorrhage lesions suggested of CNS vasculitis. In addition to diffuse brain parenchymal involvement as assessed by MRI of the brain, irregular narrowing of V4 segment of right vertebral artery was described in our patient. To the best of our knowledge, vertebral artery involvement in primary systemic vasculitis was infrequent reported, as described in one Chinese girl diagnosed Takayasu arteritis (18), and in those with childhood MPA, vertebral artery involvement was even rarer.

**Table 1:** Reported cases of childhood MPA with CNS manifestations (*n* = 7).

**Reference**	**Publication year**	**Country**	**Age (Y)**	**Sex**	**Symptom**	**Brain Imaging**	**Treatment**	**Outcome**
Hattori et al. (3)	2001	Japan	N/A	N/A	Seizure	N/A	N/A	N/A
Tan et al. (4)	2013	Brunei	10	F	Seizure	CNS hemorrhage	CYC, MP, PLEX	Survived
Iglesias et al. (5)	2013	United Kingdom	12	F	Headache Visual loss	CNS hemorrhage	CYC, P	Survived
Sun et al. (6)	2014	China	13.8	F	Dizziness Seizure	Focal ischemia	CYC, MP, PLEX	Death
Sun et al. (6)	2014	China	13.3	F	Seizure	N/A	CYC, MP, PLEX, IVIG	Death
Basu et al. (7)	2015	India	4.9	F	Seizure	N/A	RTX, MP, PLEX	Survived
Bhadu et al. (8)	2016	India	14	F	Seizure	CNS hemorrhage	CYC, MP	Survived

*CNS, central nervous system; CYC, cyclophosphamide; F, female; IVIG, intravenous immunoglobulin; MPA, microscopic polyangiitis; MP, methylprednisolone; N/A, not applicable; P, prednisolone; PLEX, plasmapheresis; RTX, rituximab; Y, year*.

In adult patients with AASV (19), cardiovascular involvement, particularly pericardial involvement, is quite uncommon (13). Iudici et al. (13) reported that only 4% patients with childhood GPA showed cardiovascular involvement, and in those with childhood MPA, cardiovascular involvement was even rarer. Despite hospitalized patients with MPA showing typical signs and symptoms of pulmonary and renal involvement, such as edema and dyspnea, myocarditis-related heart failure can mimic the symptoms of pulmonary and renal involvement. Thorough investigations are accordingly highly recommended to screen for rare organ manifestations. The natural history of the disease, which has a higher recurrence rate in childhood MPA (16), and prednisolone tapered dose could presumably explain the cause of cardiovascular manifestation and new-onset seizure in our case.

Treatment should be promptly initiated in case of patients with severe MPA and organ- or potentially life-threatening disease. Based on the most recent European League Against Rheumatism (EULAR) (20) and Childhood Arthritis and Rheumatology Research Alliance (CARRA) recommendations (21), the overall treatment plan can be divided into two stages: induction remission and maintenance therapy. In terms of induction remission therapy, intravenous administration of CYC combined with MP is consistently recommended by all guidelines for at least 3 to a maximum of 6 months, which was followed in the present case.

As evident from Table 2, differential diagnosis of new-onset CNS symptoms, even if only infrequently observed, should be open to all possibilities based on life-threatening organs and particularly curable ones. To ensure prompt diagnosis and management, it is imperative for physicians to increase their awareness, which can be achieved by thorough physical examination and screening for unusual organ involvement.

**Table 2:** Learning points.

**The present case with MPA revealed rapidly progressive glomerulonephritis without neurological manifestation**.
**Differential diagnosis of new-onset neurological manifestations contains infection according to the immunocompromised status of this patient, posterior reversible encephalopathy syndrome, and CNS vasculitis as a result of the disease itself**.
**Before concluding the diagnosis and arranging the investigation, a thorough physical examination, particularly a neurological evaluation, is recommended**.
**Despite being scarcely reported, CNS vasculitis is organ threatening, and involvement of multidisciplinary care teams is highly advisable**.

*CNS, central nervous system; MPA, microscopic polyangiitis*.

## Data Availability Statement

The raw data supporting the conclusions of this article will be made available by the authors, without undue reservation.

## Ethics Statement

The studies involving human participants were reviewed and approved by the Institutional Review Board, Faculty of Medicine Vajira Hospital, (COE no. Si 018/2021x). Written informed consent to participate in this study was provided by the participants' legal guardian/next of kin.

## Author Contributions

PS contributed to the literature review, preparing the figures, and drafting the manuscript. SW drafting the manuscript and initiating the tables. VL and OS contributed to the patient care and helped in manuscript approval. ST contributed to the patient care, literature review, preparing the figures, and revising the manuscript. All authors contributed to the article and approved the submitted version.

## Conflict of Interest

The authors declare that the research was conducted in the absence of any commercial or financial relationships that could be construed as a potential conflict of interest.

## Publisher's Note

All claims expressed in this article are solely those of the authors and do not necessarily represent those of their affiliated organizations, or those of the publisher, the editors and the reviewers. Any product that may be evaluated in this article, or claim that may be made by its manufacturer, is not guaranteed or endorsed by the publisher.
