# Supplementary material for: Atypical Neurological Manifestation in Childhood Microscopic Polyangiitis: A Case Report and Review of Literature
Source: Front Pediatr. 2022 Mar 11;10:855338. doi: 10.3389/fped.2022.855338 (PMC8963201; doi:10.3389/fped.2022.855338)
Supplement: Supplementary file 1 [file Table_1.docx]

**Supplementary Figure 1.** The schematic overview of the articles on MPA patients with CNS involvement. CNS: central nervous system, MPA: microscopic polyangiitis

Full text articles included

(n=6)

Full text articles excluded, with reasons

(n=38)

30 with no CNS vasculitis patients

6 with no data regarding CNS outcome

2 with review articles

Full text articles assessed

(n=44)

Records screened

(n=95)

Records excluded, with reasons

(n=51)

21 with no MPA patients

16 with review articles

7 with guideline

4 with adult data

3 with non-English language

Records identified through database searching

(n=94)

Additional records identified through other sources (manual search from reference lists of relevant articles)

(n=1)
